# Lipid Cargo of Small Extracellular Vesicles as a Novel Contributor to Cardiovascular Diseases

**DOI:** 10.3390/ijms27188431

**Published:** 2026-09-21

**Authors:** Cameron Keene, Lacey Knudsen, Lucy Liaw, Ilka M. Pinz

**Affiliations:** 1 Center for Molecular Medicine, MaineHealth Institute for Research, Scarborough, ME 04047, USA; cameron.keene@mainehealth.org (C.K.); lucy.liaw@mainehealth.org (L.L.); 2 Department of Medicine, Tufts University School of Medicine, Boston, MA 02155, USA

**Keywords:** extracellular vesicles, lipidomics, lipid species, cardiovascular disease

## Abstract

Cardiovascular diseases (CVDs) are the most prevalent cause of mortality globally, despite decades of targeted treatment of known risk factors including hypercholesterolemia. This review summarizes recent work on other lipids beyond cholesterol that may also contribute to CVD and serve as novel therapeutic targets. In particular, work on extracellular vesicles (EVs), which are membrane-delimited particles released by many cell types, suggests that lipid classes such as sphingolipids, phospholipids, and glycosylated lipids may contribute to CVD progression. EVs are particularly interesting due to their high biocompatibility, specific targeting, and diverse payloads. They enable intercellular communication through the transfer of lipids, proteins, and nucleic acids. Changes in EV lipid composition are highly dependent on the cellular environment, potentially enabling their use as biomarkers. While most of the published body of work on EVs is focused on protein and miRNA cargo, recent advances in metabolomic techniques enable the probing of changes in distinct EV lipid profiles associated with CVD. Herein, we summarize EV lipid work of the last five years that reaches beyond cholesterol and triglycerides and describe known lipid function in EVs and CVD. More research is needed to determine exactly how changing lipid loads of EVs impact the cardiovascular system, and how EVs and their selective lipid cargo may offer a novel approach for the treatment and diagnosis of CVD.

## 1. Introduction

The spectrum of cardiovascular diseases (CVDs) remains the leading cause of global mortality and morbidity, despite improved treatments and acute care advances [1]. The continued high incidence of CVD strongly suggests that there are other so-far-unknown contributing factors that require further investigations to advance our understanding of disease initiation and progression. Recent work indicates that lipids such as sphingolipids, phospholipids, and inflammatory lipids contribute to cardiovascular disease through partly distinct but intersecting mechanisms such as lipoprotein retention and aggregation, endothelial dysfunction, maladaptive vascular inflammation, foam-cell formation, smooth muscle remodeling, and impaired inflammation resolution. The strongest recent evidence centers on ceramides and sphingomyelins in atherosclerosis and on oxylipin imbalance as a determinant of persistent arterial inflammation [2,3,4,5]. Sphingomyelins and ceramides appear to act early in lesion initiation by altering lipoprotein behavior in the arterial wall. Atherosclerosis begins with intimal lipoprotein retention, and sphingomyelin hydrolysis within LDL generates ceramide-rich particles that aggregate more readily, prolong residence in the intima, and promote plaque initiation [2,6,7,8]. Ceramide accumulation also drives endothelial dysfunction, foam-cell formation, LDL aggregation, and vascular smooth muscle cell phenotype changes, linking early retention to later plaque growth and instability [2,3]. Phospholipids contribute to cardiovascular disease through lipoprotein organization, changes in membrane biophysics, and downstream bioactive lipid generation more than through one uniform pathogenic pathway. Plasma lipoproteins are surfaced by phospholipids, which regulate apolipoprotein and enzyme binding and thereby influence lipoprotein metabolism, a key upstream step in subendothelial retention and atherogenesis [9]. Recent lipidomics further suggests that specific phosphatidylcholine, phosphatidylethanolamine, lysophosphatidylcholine, and lysophosphatidylethanolamine patterns track poor cardiovascular health and cardiometabolic remodeling rather than a single universal CVD signature [10]. Inflammatory lipids derived from membrane polyunsaturated fatty acids (PUFAs) help determine whether vascular inflammation resolves or becomes chronic. Eicosanoids and other oxylipins are generated from phospholipid-derived PUFAs by cyclooxygenases, lipoxygenases, and cytochrome P pathways, and they regulate leukocyte recruitment, vascular tone, inflammatory amplification, and tissue repair in the vessel wall [4,11,12]. In atherosclerosis, progression appears to reflect a shift away from balanced initiation-and-resolution signaling toward persistent production of pro-inflammatory mediators and insufficient resolution [4,5,13].

Taken together, the current evidence supports that certain lipid classes contribute to CVD initiation and progression. Interestingly, an emerging area of research concerns extracellular vesicles (EVs), which provide a new mechanism of communication between organ systems. This is a developing area of research that may provide insights into additional lipid-mediated mechanisms that contribute to CVD.

EVs are cell-derived, membrane-delimited particles that are released by platelets, erythrocytes, leukocytes, endothelial cells, cardiomyocytes, and adipose tissues. EV cargo and lipid membrane composition varies with CVD status [14,15,16,17,18,19]. There are strong indications that EVs are non-invasive biomarkers and functional mediators in atherosclerosis, myocardial infarction, heart failure, hypertension, and cardiac hypertrophy [14,15,16,17,18,19]. Furthermore, given their genesis from cellular cytoplasm and membranes, EVs are rich in proteins, various RNA species, and membrane lipids. Because of these qualities, EVs serve as systemic signaling conduits.

**Figure 1:**
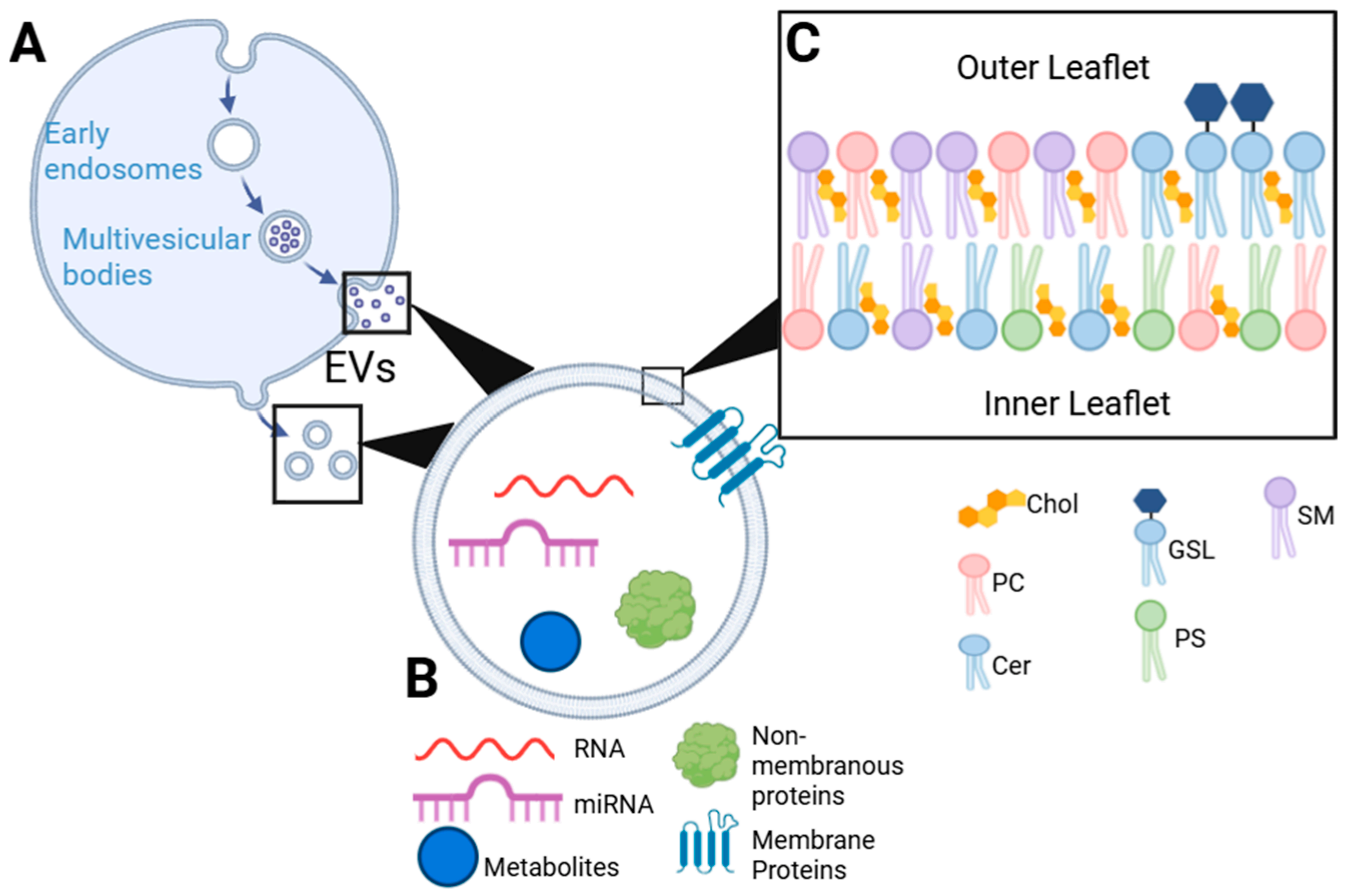
Overview of EV biogenesis and general composition. (**A**) EVs are primarily derived through two pathways, resulting in distinct EV subpopulations known as exosomes or ectosomes. Exosomes form via endocytosis of the plasma membrane, formation of multivesicular bodies where cargo loading occurs, and released by exocytosis. Ectosomes are formed from the outward budding of the plasma membrane. (**B**) A generalized view of EV structure. EVs are membrane-delimited particles, which can transfer various RNAs, miRNAs, metabolites, proteins, and lipids as intercellular communicators. (**C**) Lipids found commonly within the membranes of EVs, which may contribute to CVD progression or resolution. Because EVs are derived from the plasma membrane, their lipid composition often reflects that of their parent cells. Cer = Ceramide, Chol = Cholesterol, GSL = Glycosphingolipid, PC = Phosphatidylcholine, PS = Phosphatidylserine, SM = Sphingomyelin. Created in BioRender. Cameron Keene. https://BioRender.com/shab903 (accessed on August 1 2026).

Classifying and naming EVs has been historically accomplished by size, wherein exosomes are relatively small in size, ranging from ~30 to 100 nm, ectosomes or microvesicles are larger, ranging from ~100 to 1000 nm, and apoptotic bodies are the largest, at 1000–5000 nm. According to the International Society for Extracellular Vesicles and their most recent consensus work, Minimal Information for Studies of Extracellular Vesicles (MISEV) [20], the classification based on size alone is insufficient. MISEV2023 emphasizes that exo- and ectosomes refer to cellular processes related to particle release and should not be used without demonstration of origin. Specifically, exosomes are the result of endosomal processes prior to release while ectosomes or microvesicles are the result of outward plasma membrane budding. Small (sEV) and large (lEV) are heavily used in the literature and will be using in this review, although exact cut-offs for sEV and lEV have yet to be determined (Figure 1).

While many studies have been published about the paracrine and endocrine functions of EVs, they are predominantly focused on protein and miRNA cargo of EVs [21,22,23,24,25]. However, there is evidence that the lipid cargo of EVs plays an important role in transporting lipid species between organ systems and that this novel mechanism of lipid transport and signaling may contribute to CVD. Advancement in this area has been aided by improved mass spectrometry approaches for the identification and classification of lipids and other metabolites. High-coverage lipidomics of EVs mainly relies on LC–MS/MS, enabling quantification of total lipid content, species composition, and sample purity [26,27,28,29,30,31,32,33]. Targeted and untargeted approaches reveal EV-specific lipid sorting relative to parent cells or tissues, as shown in adipose tissue, protozoa, and mammary epithelium [26,27,28,30]. Improvements in the ability to characterize lipids have allowed for a deeper understanding of their roles in biological processes of health and disease. Today, this understanding goes above the well-established role cholesterol and triglycerides play in CVD [29]. However, pitfalls include co-isolation of lipoproteins and non-EV lipid particles, ion suppression, and incomplete structural annotation of lipids. Efforts to standardize lipidomic approaches are underway, and adherence to the Lipidomics Standards Initiative guidance is being strongly advised [16,29,33,34]. Additionally, the use of different upstream EV isolation methods—ultracentrifugation, density gradients, size exclusion chromatography, microfluidics, or affinity capture—critically affects lipid readouts and comparability across studies [16,19,33,34]. Nevertheless, lipids are perhaps the most diverse group of biomolecules, and have a major role in biological homeostasis.

Data published within the last 5 years demonstrate that many sphingolipids and phospholipids are frequently altered in CVD or other disease processes linked with CVD progression, such as obesity and diabetes. Some of these lipid species are commonly enriched in EVs when compared to their parent cell under healthy conditions, and this enrichment can be disrupted in disease states. Given the relatively new interest in lipid classes and species that are different from cholesterol and triglycerides combined with broader challenges in lipidomics, exactly how these lipids may contribute to CVD progression or treatment and EV function often remains unknown. Herein, select lipid classes that are emerging as potential drivers or biomarkers of CVD and their overlapping expression in EVs will be discussed. EVs as therapeutic opportunities in CVD, current limitations in EV and lipidomic research, and possible solutions to those limitations conclude this review.

## 2. Lipids in the Bilayer of Extracellular Vesicles

The lipid bilayer of EVs is composed of various lipid species and can reflect the lipid composition of the cells of origin, with adaptations to allow for the small size and diverse cargo of the EV [35,36]. Additionally, disease states contribute to changing the ratio of different lipid classes in EVs. This includes obesity-induced changes in lipid composition of exosomes in rats [37], the increased conversion of sphingosine to sphingosine-1-phosphate (S1P) in EVs derived from ovarian cancer patients [38], changes in lipid composition in brain-derived EVs in deceased Alzheimer’s patients versus deceased patients without Alzheimer’s diagnoses [39], and EVs derived from adipocytes with different lipid compositions when exposed to vitamin D and TNF alpha in inflammatory conditions [35]. A summary of lipids within EVs with a link to CVD is presented in Table 1.

### 2.1. Cholesterol

Cholesterol is ubiquitous in cell and EV membranes. Studies using multi-omics techniques have validated that cholesterol is highly abundant in EVs, ranging between 40% and 60% of total lipids in EVs derived from human blood plasma [58]. Cholesterol is tightly linked with membrane fluidity and organization of cell membranes. Disrupting cholesterol levels in cellular membranes causes abnormal membrane function and an altered immune response [40]. The effect of cholesterol via LDL, VLDL, and HDL particles on cardiovascular disease and human health has been thoroughly studied, so cholesterol is not a primary focus of this review. However, it would be inappropriate not to mention the role of cholesterol in the context of EVs.

One study [41] demonstrated that sEVs have an enriched cholesterol content in vivo compared to cellular plasma membranes. Cholesterol associates with the headgroups of phospholipids, acting to fill the spaces between them. This association also increases the ordering of the lipid tails, resulting in a membrane that is thicker and more tightly packed [40]. This membrane characteristic is important because an increase in the packing density of lipids likely allows EVs to maintain a smaller relative size and enables more efficient retention of cargo.

The concentration of cholesterol in the plasma membrane of the cells of origin impacts the release of sEVs from these cells. Indeed, this was observed in an in vitro study of astrocytes, where increased plasma membrane concentrations of cholesterol reduced the release of sEVs [42]. Although this study utilized neuronal cells, it demonstrated that the cholesterol-dependent release of sEV is regulated by the PI3K/Akt pathway. The PI3K/Akt pathway has a broad relevance to diabetes and cardiovascular disease, suggesting that varying the number of EVs released may be a contributing mechanism in these prevalent pathologies.

### 2.2. Sphingolipids

#### 2.2.1. Sphingomyelins

Sphingomyelins (SMs) are a class of sphingolipids abundantly localized in mammalian cell membranes. Consequently, SM is one of the most enriched lipid components of EVs [43]. Due to their shape and biophysical properties, SMs are crucial in creating membrane curvature in cell membranes, particularly due to homeostatic balance with ceramides. While SMs have no innate curvature, actions of sphingomyelinases release phosphocholine from SM to produce ceramides. Unlike SMs, ceramides have an innately negative curvature, encouraging the inward budding of plasma membranes and the formation of endosomes. Therefore, SM is an essential lipid that enables the tightly curved membranes seen in sEVs when converted to ceramide [59].

SM also regulates protein p24 [60], which is important in vesicle budding from the Golgi apparatus, suggesting a modulating role in EV biogenesis. There is compelling evidence that SMs in human plasma-derived EVs are enriched immediately after ST-segment elevation myocardial infarction when compared to EVs of subjects with a matched cardiovascular risk and demographics but without CVD [61]. This enrichment decreased 24 h after the onset of reperfusion. Additionally, in this study, the increase in SM lipids in EVs correlated with peak levels of troponin and inversely correlated with the ejection fraction of patients, suggesting that an increased SM cargo in EVs may have a negative effect on cardiac function.

Various circulating SMs in humans have been implicated in different types of cardiovascular disease. This variance, in part, is dependent on the length of the fatty acyl sidechains. A study of Chinese adults showed that a two-fold increase in circulating SMs containing palmitic acid (16:0) was associated with a 50% increase in cardiovascular mortality risk [62], suggesting that shorter-chain SMs are associated with negative health outcomes. A study investigating 12 Native American populations [45] in the United States found an association between all-cause mortality and circulating SMs and ceramides with 16:0 fatty acid length chains. Interestingly, longer-length acyl sidechains between 20:0 and 24:0 in both SMs and ceramides were inversely correlated with mortality, supporting the evidence that the chain length of these lipids is an important factor determining cardiovascular disease outcomes. Importantly, according to experimental data within the EV database ExoCarta [63], SMs with fatty acyl chain lengths of 16:0, 22:0, and 24:0 are present in sEVs, supporting the conclusion that the EV SM composition is potentially a significant contributor to CVD.

#### 2.2.2. Sphingosin-1-Phosphate

Sphingosine-1-phosphate (S1P) is a bioactive sphingolipid that functions as both an intracellular signaling molecule and an extracellular ligand for S1P receptors 1–5 (S1PR1–5), with particularly important effects on endothelial integrity, vascular tone, inflammation, angiogenesis, cardiomyocyte function, and cardiac remodeling [64,65]. S1P signaling is also intimately linked to EV biology by activation of G_i_-protein-coupled S1P receptors on multivesicular endosomes, which promotes endosomal maturation and the sorting of proteins into intraluminal vesicles destined for EV release. This suggests that sphingolipid metabolism can regulate EV biogenesis and cargo composition [66,67]. Although much of the mechanistic work establishing this pathway predates the past five years, recent reviews continue to identify S1P-dependent signaling as an important component of sphingolipid-mediated EV formation and cargo sorting. Circulating S1P is associated with hypertension, inflammatory and cardiovascular disease biomarkers, while altered S1P signaling has been implicated in atherosclerosis, myocardial infarction, ischemia/reperfusion injury, and heart failure [18,68]. Consistent with a cardioprotective role, experimental studies demonstrate that S1P signaling through S1PR1 can reduce myocardial injury and improve outcomes following myocardial infarction, while platelet-derived S1P has been shown to contribute to cardioprotection during acute myocardial infarction [67,69]. Thus, the intersection of S1P metabolism, S1P receptor signaling, and EV biology represents a potentially important mechanism for regulating intercellular communication during cardiovascular stress and may provide opportunities for developing EV- or S1P-based biomarkers and therapeutic strategies for cardiovascular disease [64,70].

#### 2.2.3. Ceramides

Ceramides are one of the most studied lipids in CVD following cholesterol and triglycerides. Composed of a sphingosine backbone and a fatty acyl side chain of varying lengths, ceramides also serve as a launching point for more complex sphingolipid synthesis via modification of their hydroxyl headgroup. Ceramides are of particular interest because they are present in cellular membranes as well as in EVs and are linked to a broad range of negative outcomes within the cardiovascular system as discussed below.

The composition of ceramides in EVs is variable, which should not be surprising given the broad heterogeneity of cell types they are released from. Payet et al. [35] demonstrate that the size of an EV released from cultured 3T3-L1 cells is directly correlated with its ceramide content. In their study, they show that small EVs are composed of a relatively higher percentage of ceramides as compared to large EVs (11.53% vs. 4.62%). A previous review from Skotland et al. [71] compared the composition of prostate EVs to other extracellularly released particles, showing only a small enrichment of ceramides. However, they call for a more thorough interrogation of the EVs from different cell types to evaluate this trend, as ceramides are implicated in the formation and release of EVs.

Specifically, neutral sphingomyelinase 2 (nSMase 2), which removes the phosphocholine headgroup from sphingomyelins to form ceramides, selectively alters the membrane structure when active. Trajkovic et al. [72] demonstrated that pharmacological inhibition of nSMase 2 with GW4869 decreases the number of EVs released from Oli-Neu cells, a model of mouse oligodendrocytes. The activity of nSMase 2 encourages the spontaneous formation of interluminal vesicles in endosomes due to the negative membrane curvature induced by ceramides, which may eventually be released as exosomes. Menck et al. [73] have verified the work of Trajkovic et al. [72], going even further to demonstrate that inhibition of nSMases causes the release of more EVs ranging from 100 to 150 nm while release of EVs ranging from 50 to 100 nm was limited. SKBR3 cells, a model for breast cancer, show that the larger EVs released under GW4869 exposure originate from the plasma membrane, have an increased expression of Wnt5A, and have a drastically higher sphingomyelin content. These results suggest that the cellular lipid content can alter the route through which EVs are released, and that the EV lipid content modulates EV payloads. Further investigations of alterations in cellular lipid handling affecting the EV payload are needed in more CVD-relevant cell lines and animal models, as the varied cell types used to investigate these effects appear to follow a similar trend.

**Figure 2:**
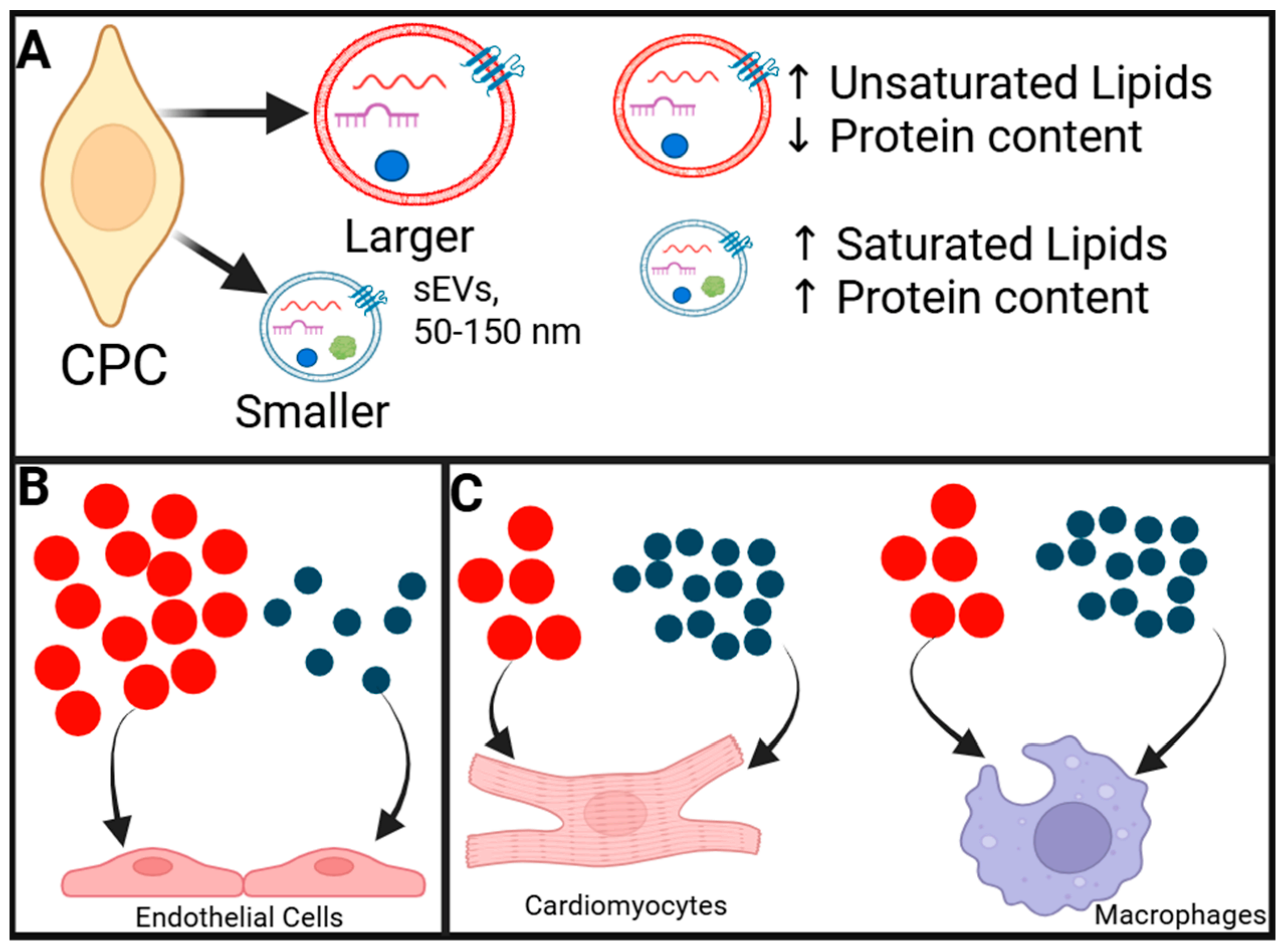
EVs released from the same cell population have dynamic cargoes and targeting abilities. (**A**) Cardiac progenitor cells (CPCs) release many EVs within the sEV (blue) range (50–150 nm). Further divisions of this sEV population by size reveals that the cargoes are not consistent across the entire size range. (**B**) Endothelial cells show preferential uptake of larger EVs (red) and limited uptake of smaller EVs. (**C**) Cardiomyocytes and macrophages show preferential uptake of smaller EVs and limited uptake of larger EVs. Created in BioRender. Cameron Keene. https://BioRender.com/shab903 (accessed on August 1 2026).

Through the studies above, it becomes apparent that the lipid content can modulate the size, release, and protein content of EVs. Even within the relatively narrow range of 50–150 nm used as one working definition for sEVs, a broad heterogeneity in size exists. The relationships between size and function have been explored by van de Wakker et al. [36]. Cultured cardiac progenitor cells were shown to release three distinct populations of EVs within the 50–150 nm sEV range when isolated with size exclusion chromatography. Variations in size are expected with this isolation method based on which fractions are collected, but the differences go beyond size. For instance, the largest EVs had the lowest protein content and the highest unsaturated lipid content compared to medium and small EVs. Further, it was demonstrated that a variety of cells have differential uptake of certain EV populations. Specifically, the largest EVs were preferentially taken up by endothelial cells while medium and small particles were better at targeting cardiomyocytes and macrophages [36] (Figure 2). These results, combined with the evidence that alterations in ceramide content affect both the release and size of EVs, indicate that approaches for treating the negative outcomes associated with ceramides must be carefully considered. It also indicates that additional research is needed to explore the interrelationships of size and function of EVs, as simply classifying EVs by size alone does not represent a comprehensive approach to their function.

Ceramides with C16:0 fatty acyl side chain lengths are directly associated with poor outcomes in CVD. This is demonstrated in the Cardiovascular Health Study [74], wherein circulating plasma levels of C16:0 and its derivative SM with C16:0 are correlated with a higher risk of sudden cardiac death in adults 65 years and older, independent of other risk factors and population characteristics. It is also noteworthy that sudden cardiac death was not linked to ceramides containing longer fatty acyl side chains, such as arachidic (C20:0) or behenic acid (C22:0) in this study. A study in a cohort of Chinese patients confirmed that ceramides with C16:0 fatty acyl side chains could potentially be used as a biomarker for acute coronary syndrome. Levels of ceramide C16:0 showed a sigmoidal correlation with risk of acute coronary syndrome above levels of 150 µmol/L, whereas levels below 150 µmol/L showed lower risk [75].

In addition to increasing general cardiovascular risk, ceramide C16:0 is known to cause endothelial disruptions. For example, patients with or without diabetes who require a below-the-knee amputation due to prolonged ischemia show ceramide C16:0 accumulation in the intima of the tibial artery. Meade et al. demonstrate that the maximally diseased areas of the intima are enriched in C16:0 ceramides regardless of diabetic status, though patients with diabetes show even higher enrichment [47]. To further understand the mechanism, the group tested the effects of ceramide C16:0 species on cultured human umbilical vascular endothelial cells. They observed that exogenous ceramide C16:0 supplementation significantly reduced proliferation and expression of protective autophagy-related proteins, while increasing rates of apoptosis.

Despite the largely negative connections presented regarding ceramides, their presence is critical to endothelial function. Three ceramide species in particular, Cer-16:0, 24:0, and 24:1, regulate vasorelaxation. Local loss of ceramide production through the deletion of SPTLC2 (serine palmitoyl transferase long chain 2), a member of the serine palmitoyl transferase complex (SPT) responsible for de novo ceramide synthesis, in mice shows significant impairment of vascular relaxation responses to blood flow and chemical or protein agonists, such as S1P and vascular endothelial growth factor (VEGF). Supplementation of ceramides 16:0, 24:0, and 24:1 was able to recover these responses, but with variable effects based on dose and species [46]. Inhibition of the SPT complex in endothelial cells via neurite outgrowth inhibitor A/B (Nogo-A/B, Reticulon 4) has been observed in wild-type C57BL/6J mice when maintained on a high-fat diet. Endothelial-cell specific deletion of Nogo-A/B (Reticulon 4) recovered ceramide levels and improved glucose tolerance and vessel contractility usually associated with obesity [76]. These results demonstrate that ceramides are tightly regulated, and further characterization of the effects of different ceramide species and their metabolism may potentially provide other avenues for treatment of cardiovascular disease.

#### 2.2.4. Glycosphingolipids

Glycosphingolipids (GSLs) are a diverse class of glycolipids with a broad distribution in human cell membranes and are particularly present at high levels in the brain. Ceramides serve as the base lipid for GSL synthesis, which are then further modified with various glycans. GSLs compose anywhere between <5% and >20% of the total plasma membrane lipids in vertebrate cells, with different GSL species being enriched in different cells. In the plasma membrane, GSLs are not homogenously distributed but assemble in microdomains called lipid rafts, where they have two major roles: to communicate extracellularly and to modulate protein activity intracellularly [48]. A specific subset of lipid rafts known as caveolae are the primary cause of these activities. Caveolae are small invaginations of the plasma membrane that are formed and stabilized by cavin- and caveolin family proteins. Specific recruitment of these proteins is enabled by the enrichment of cholesterol, sphingolipids, and GSLs [49]. Endocytosis of caveolae and later release of exosome-like sEVS has been observed in endothelial cells stimulated with fatty acids, indicating that caveolae may be important in sEV formation and thus linking caveolae and extracellular communication [77].

**Figure 3:**
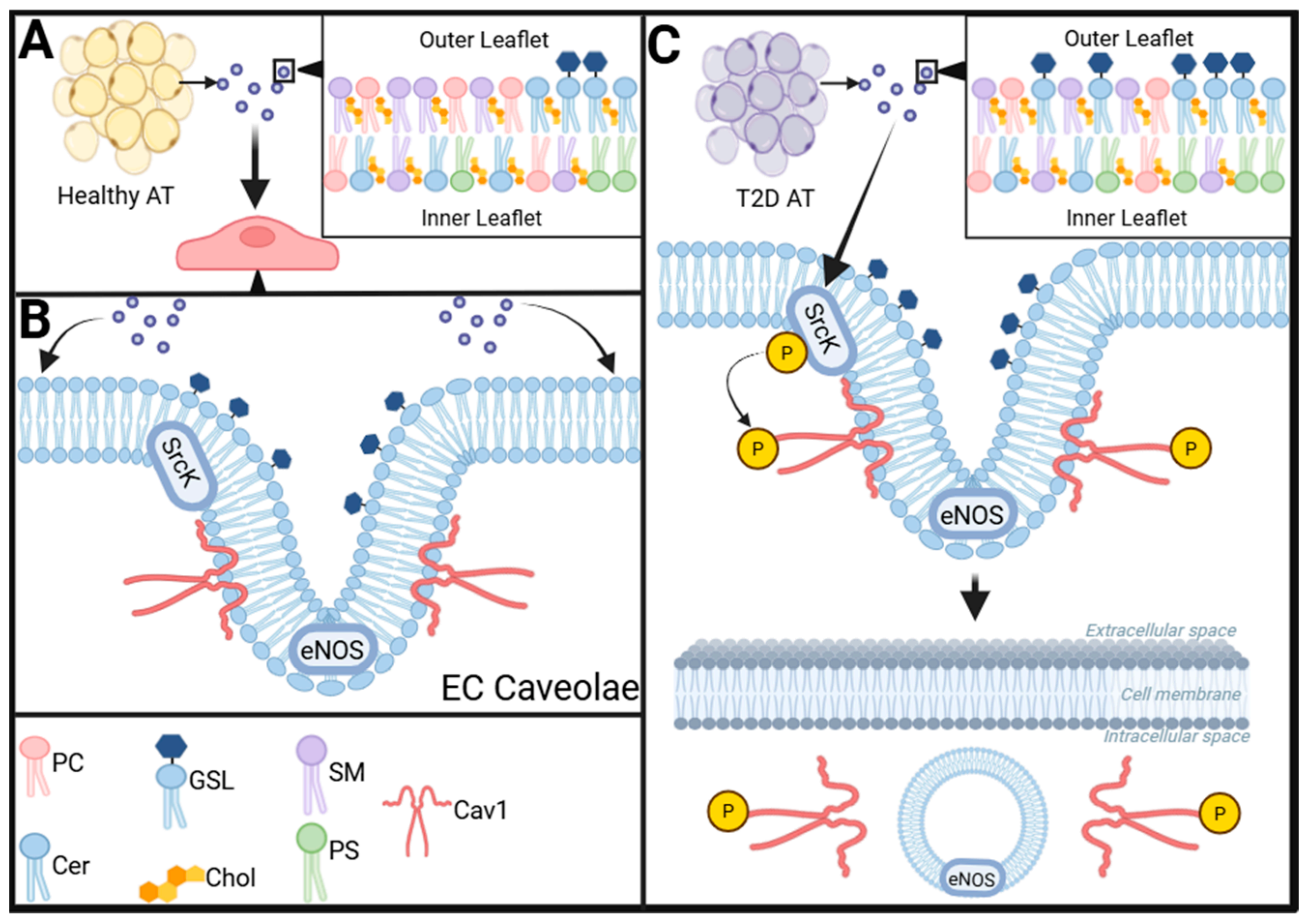
Disease states can alter EV lipid composition, changing their route of uptake and downstream effects. (**A**) EVs isolated from adipose tissue (AT) biopsies from lean, healthy human controls have lipid profiles that reflect the state of their parent cells. Endothelial (EC) cells are then able to uptake these EVs. (**B**) ECs have many caveolae, which host proteins critical to their normal functions, such as endothelial nitric oxide synthase (eNOS). Caveolae are small membrane invaginations stabilized by Caveolin 1 (Cav1), which localizes to areas of the plasma membrane enriched in glycosphingolipids (GSLs). EVs from healthy patients show limited localization to caveolae and maintain the normal function of these structures. (**C**) AT biopsy-derived EVs from obese type 2 diabetic (T2D) patients are greatly enriched in GSLs. These EVs show enhanced localization to caveolae, which causes Src kinase (SrcK) to be phosphorylated. SrcK then phosphorylates Cav1, leading to disassociation of Cav1 from the membrane. Loss of Cav1 collapses caveolae, which initiates the internalization of eNOS, limiting its activation and leading to endothelial disruption. Adapted from Mirza et al. [50]. Cer = Ceramide, Chol = Cholesterol, GSL = Glycosphingolipid, PC = Phosphatidylcholine, PS = Phosphatidylserine, SM = Sphingomyelin. Created in BioRender. Cameron Keene. https://BioRender.com/shab903 (accessed on August 1 2026).

Like ceramides, GSLs accumulate in atherosclerotic plaques of the aortic walls [78]. Additionally, in a hypertrophic mouse model, inhibition of GSL synthesis improved cardiac function and reduced oxidative stress [79]. This implies that increased GSLs in cardiomyocytes may be related to cardiovascular disease and cardiomyocyte hypertrophy. Gb3, GD1, GM1, GM3, hexosylceramides, and lactosylceramides are upregulated in EVs isolated from adipose tissue biopsy cultures of obese Type-2 diabetic patients compared to lean healthy controls. Application of the diseased EVs to human adipose microvascular endothelial cells showed enhanced localization to caveolae, which induced significant alterations of these structures through Caveolin 1 and Scr kinase phosphorylation. This is particularly important in the context of CVD, as caveolae host endothelial nitric oxide synthase (eNOS) and mechanotransduction proteins critical to normal endothelial cell function [50] (Figure 3).

There is evidence that HexCers and other GSLs are enriched in EVs compared to the cells of origin [71]. Using Time-of-Flight Secondary Ion Mass Spectrometry (TOF-SIMS), Skalska et al. [80] analyzed the lipid composition of ß-cells and small/large EVs when exposed to normoglycemic, elevated glycemic, and hyperglycemic conditions. Expression of total HexCer species was not significantly altered between conditions in ß-cells. Compared to parental cells, EVs were consistently enriched in HexCers 42:1 and 36:1 while showing decreases in 38:0, 38:1, 38:2, and 40:2 species across all conditions. Of note, hyperglycemic conditions induced even greater enrichment of HexCer 42:1 in sEVs, and increased or decreased HexCer 36:1 and 36:2 respectively in both EV populations. Under normoglycemia, GSL levels between ß-cells and EVs are stable and reflect one another. Unlike HexCers, GSL species in ß-cells and EVs are significantly more sensitive to high-glucose environments. Substantial reductions in the phosphosphingolipid fragment ([C_2_H_9_NPO_4_]+, *m*/*z* 142.07) and enrichment of GSL ions ([C_42_H_81_NO_3_Na]+, *m*/*z* 750.49) and ([C_47_H_91_NO_9_Na]+, *m*/*z* 836.80) were seen in ß-cells and both EV populations, though the enriched species were more prevalent in EVs, and even more so in sEVs. These results, along with those of Mirza et al. [50], indicate that EV lipid composition is highly dynamic and a possible driver of CVD.

Hexosylceramides (HexCer) are a type of glycosphingolipid where a hydrophilic hexose (glucose or galactose) is attached to a ceramide backbone [81], and they are linked to cardiovascular and metabolic diseases. In a lipidomic study [82], elevated diastolic blood pressure was correlated with circulating HexCer species (22:0, 24:0 and 24:1) in plasma samples taken from Mexican American families. In a study of overweight obese women [83], HexCer were inversely correlated with BMI and total cholesterol, and directly correlated with HDLc, suggesting that low HexCer may have a role in cardiometabolic disease. Most of the HexCer in this study demonstrated this relationship, while Hex2Cer 18:1/14:0, Hex2Cer 18:1/16:0, and HexCer 18:1/20:0 showed the strongest correlation. Three other HexCer had very low concentrations and did not show any changes for obese women versus women with a normal body weight. The impact of HexCer is further explored in a study of 24 patients with coronary artery disease, of which half also had T2D. In this study, nine different species of HexCer were downregulated in patients with DM compared to those without DM, with HexCer-d18:1–23:1(2-OH) being downregulated the most [81]. Further studies to evaluate the HexCer composition of EVs from different cell sources are needed to fully understand their contribution to CVD. In particular, investigating the regulation mechanisms of HexCer in different disease states, such as diabetes and obesity, is needed.

### 2.3. Phosphatidylserine

Phosphatidylserine (PS) is a negatively charged phospholipid that resides on the cytosol-facing leaflet of the plasma membrane in normal physiological conditions. PS is rarely found on the cell surface; it moves to the outer layer of the plasma membrane to induce apoptosis, to signal macrophages for cell disposal, and to activate platelets for coagulation by using a scaffolding system that allows coagulation factors to aggregate [51]. EVs extracted from human urine, saliva, breast milk, and semen are highly enriched with PS in the outer membrane leaflet. Of interest, EVs from blood samples were found to be nearly devoid of PS [55,84]. Because of the previously mentioned role in supporting the clotting cascade, it would follow that under normal conditions, exposure of PS in blood EVs should be limited to prevent errant clotting.

**Figure 4:**
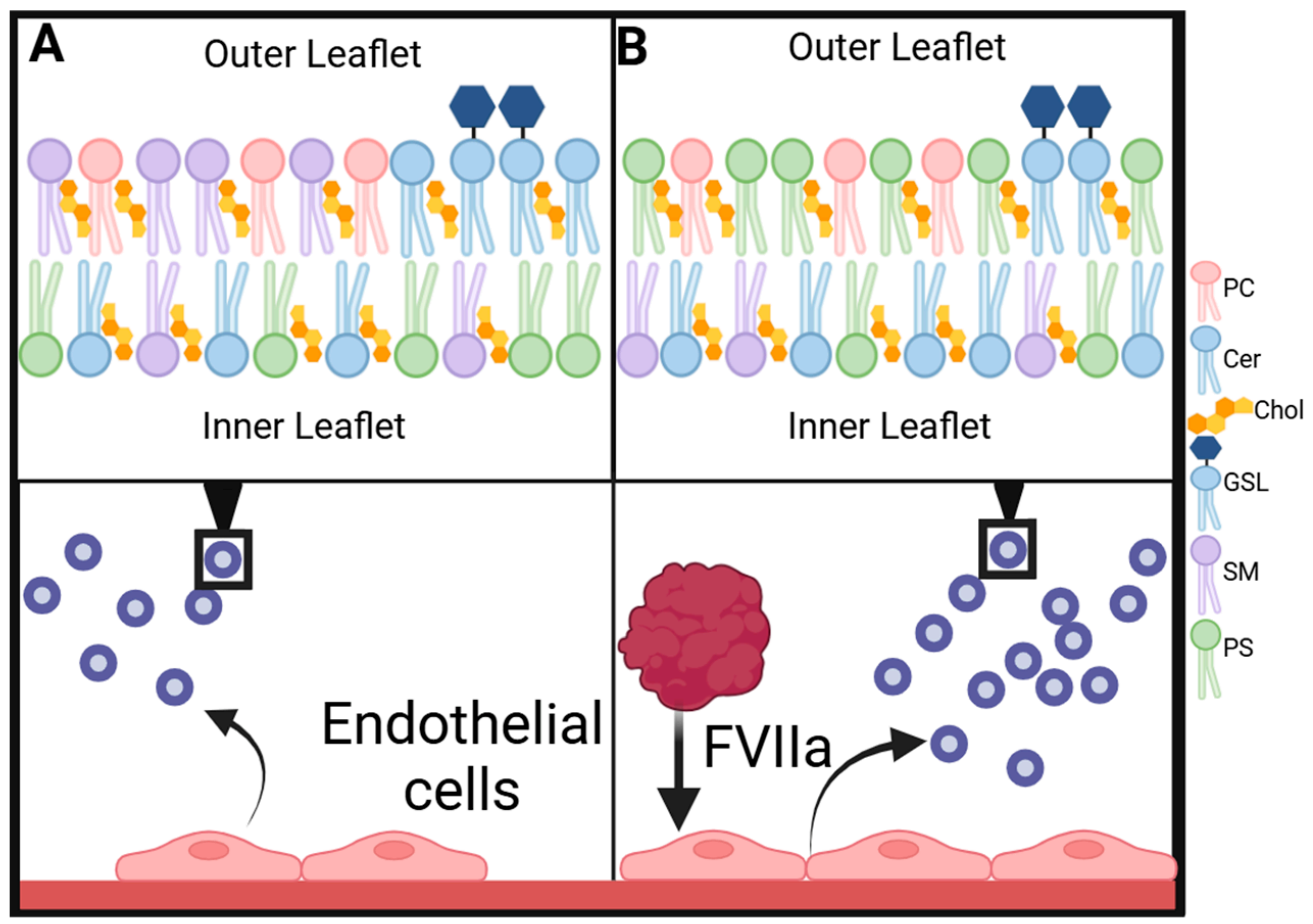
Normal physiological stimuli alter EV lipid distribution and count. (**A**) EVs released from endothelial cells (ECs) under normal conditions maintain phosphatidylserine (PS) on the inner leaflet. Most circulating EVs demonstrate low PS externalization. (**B**) ECs stimulated with activated Factor VII (FVIIa) during the clotting cascade release a higher number of EVs compared to basal conditions. These EVs externalize PS to the outer leaflet, which can further promote clotting activities. Cer = Ceramide, Chol = Cholesterol, GSL = Glycosphingolipid, PC = Phosphatidylcholine, PS = Phosphatidylserine, SM = Sphingomyelin. Created in BioRender. Cameron Keene. https://BioRender.com/shab903 (accessed on August 1 2026).

EVs that originate from endothelial cells via activated factor (F) VII (FVIIa) are highly enriched in PS and particle count compared to EVs released during basal conditions. FVIIa is linked to the externalization of PS in the plasma membrane and in the biogenesis of EVs. Changes in PS localization and EV release under FVIIa exposure were observed to depend on EPCR (Endothelial Protein C Receptor) binding and subsequent PAR1 activation, as loss of either protein abrogated the effects of FVIIa [53] (Figure 4). EVs originating from FVIIa activity have higher procoagulant activity, potentially due to the negative charge on PS [85]. PS-presenting EVs also contribute to tissue homeostasis. In vitro, Mardin-Darby canine kidney II (MDCKII) epithelial cells undergoing apoptosis released EVs basally. The formation of these EVs was due in large part to re-localization of PS to the outer membrane, which imparts curvature away from the extruding cell and cell layer. These EVs were taken up quickly by neighboring cells and phagocytes [52].

PS also appears to be important in the resolution of myocardial infarction. In a mouse model of cardiac ischemic injury, PS was downregulated in injured heart tissue. Following left anterior descending (LAD) artery ligation, nine species of polyunsaturated PS were significantly decreased, with PS (40:6) being the most decreased compared to mice after sham surgery. PSS1 (phosphatidylserine synthase 1), an enzyme responsible for conversion of phosphatidycholine (PC) to PS, was decreased in mice after LAD ligation and in primary neonatal rat cardiomyocytes subjected to ischemia and glucose deprivation. Adenoviral overexpression of PSS1 in rat cardiomyocytes rescued apoptosis through a reduction in Bax expression, an increase in Bcl2 expression, and a reduction in the ratio of cleaved-caspase-3/caspase-3 [86]. This is supported by evidence that an autoantibody against phosphatidylserine was increased in patients with acute myocardial infarction and in patients with unstable angina when compared to matched subjects without CVD or other acute or chronic illnesses [87]. In a mouse model of cardiac ischemic injury, PS given orally was linked to a 50% increase in cardiomyocyte survival, a decrease in infarct size, and an improved ejection fraction without increasing apoptotic markers [54]. In the same study, PS reduced the activations of neutrophils, which cause inflammation and cardiomyocyte death after infarct. These data indicate that the role of PS in CVD goes beyond apoptotic signaling associated with membrane localization. Further investigations into the protective effects of PS are needed in humans, but may hold promise in mitigating disease.

### 2.4. Phosphatidylcholine

Phosphatidylcholines (PCs) are modified diacylglycerides, carrying a phosphocholine headgroup. Thus, they are comparable to sphingomyelins in terms of their membrane-shaping potential [55]. Changes in circulating PC species and levels have been investigated for their impacts in CVD. Rong et al. [56] evaluated the correlative value of circulating PCs and b-type natriuretic peptide levels in patients that progress to heart failure following a myocardial infarction. They found that many PC species were decreased in the progressive heart failure group, while PC (22:4/14:1) was increased. The increase in this specific PC species was significantly and positively correlated with b-type natriuretic peptide levels, indicating a potential use as a biomarker for heart failure progression. Further, Vianello et al. [88] showed that a reduction in phosphatidylcholine/phosphatidylethanolamine (PC/PE) molar ratios in plasma, where many species of both classes were decreased, was negatively correlated with an increased insulin resistance risk (HOMA-IR ≥2.5) and circulating nucleotide-binding oligomerization domain (NOD), leucine-rich repeat (LRR)-containing protein 3 (NLRP3) in overweight patients (BMI 25–29.9). NLRP3 is an indicator of metabolically induced inflammation, wherein elevated levels (>1.48 ng/mL) are associated with maladaptive cardiac remodeling responses, including increased left ventricle thickness and mass. Kvasnicka et al. [89] evaluated the circulating lipids of 50 heart failure patients. PC species esterified with long-chain polyunsaturated fatty acids (PUFAs) (PC 42:10, PC 36:6, PC 40:9, PC 37:6, PC 40:7 and PC 34:5) were significantly elevated in survivors compared to non-survivors (n = 13) [89].

While changes in PC species and levels are clearly linked to CVD, it is unclear whether these changes reflect total plasma lipids or whether EVs may contribute to these changes. Timmerman et al. [57] set out to answer this question in the context of patients undergoing carotid endarterectomy and the predicative value of a future major adverse cardiovascular event (MACE). Isolating plasma EVs, total and those that coprecipitate with LDL, and comparing their lipidomic profiles against complete plasma showed differences between non-MACE and future MACE patients. In patients who would experience MACE following the endarterectomy, PC (14:0/22:6) was decreased across all three sample types, while PC (16:0/22:5) was significantly reduced in plasma and total EV samples. The reductions in PC species of this study [57] are aligned with other studies [89,90] evaluating changes in PC in the context of disease, but it leaves the question unaddressed from which tissue these EVs originate. Kumar et al. [37] demonstrated that alterations in the ratio between PC and PE in EVs during a high-fat diet can lead to insulin resistance and systemic inflammation in male C57BL/6 mice. EVs isolated from fecal samples of mice maintained on a high-fat diet (HFD) and T2D human patients showed drastic changes in EV lipid composition compared to healthy controls for either group. In mice undergoing HFD treatment, EVs showed a significant expansion of PC while PE was severely diminished. EVs from T2D patients had expansion of both PC and PE, though PC increased more proportionally. Oral administration of EVs from either species to lean mice caused insulin resistance and glucose intolerance, though HFD EVs had a stronger effect. Mechanistically, PC-enriched EVs are preferentially targeted to liver macrophages while PC-deficient EVs are targeted to hepatocytes. Macrophages targeted by PC-enriched EVs produced more TNF-α and IL6, leading to increased systemic inflammation. While showing the opposite trend of PC distribution, these results help corroborate the work of Vianello et al. [88], showing that insulin resistance and inflammation are in part controlled by lipid signaling, especially in the case of EVs.

### 2.5. Cardiolipin

Cardiolipin (CL) is the signature phospholipid of the inner mitochondrial membrane. It enables mitochondrial cristae formation and supports respiratory chain organization and overall mitochondrial membrane integrity. While cardiolipin levels have so far not been clearly established in extracellular vesicles [91,92], cardiolipin is enriched in mitochondria-derived microvesicles. The formation of these microvesicles is primarily considered an intracellular quality-control pathway before the induction of bulk mitophagy, especially under oxidative stress, and aids in selectively removing damaged mitochondrial material [93,94,95]. However, mitochondria-derived microvesicles are also released into the circulation, and there they have clear relevance to CVD because they can promote mitochondrial dysfunction and oxidative stress in remote tissues. This has implications for myocardial infarction, heart failure, vascular dysfunction, and aging-associated cardiovascular disease [91,93,96]. In atherosclerosis, CL can have pro- as well as anti-atherosclerotic functions [97]. CL and its oxidative modifications promote atherosclerosis progression through oxidative stress, apoptosis, and inflammation. However, CL externalization to the outer mitochondrial membrane acts as a signal for mitophagy, removing dysfunctional mitochondria and safeguarding against atherosclerotic progression [97]. Recently, Swain et al. have shown that protecting mitochondria CL levels in acute myocardial infarction of swine and mouse models has the potential to limit infarct size and promote cardiac recovery [98]. These findings strongly suggest that understanding CL levels in mitochondria-derived EVs as well as potential other EVs may provide an important therapeutic approach to improve CVD outcomes. Further research into EV subclasses including mitochondria-derived EVs is needed to delineate their function in CVD.

### 2.6. Bioactive Lipids

Bioactive lipids, including arachidonic acid (AA) and its oxygenated metabolites, the eicosanoids, represent an important but relatively understudied component of the EV lipid cargo. EV membranes contain phospholipids that serve as reservoirs for AA. Additionally, EVs can carry phospholipases and enzymes involved in eicosanoid biosynthesis, allowing them not only to transport lipid mediators but also potentially to generate bioactive eicosanoids within the extracellular environment or following uptake by recipient cells [99,100]. Targeted lipidomic analyses have confirmed that EVs contain multiple polyunsaturated fatty acids and eicosanoids and that the EV lipid profile can change substantially according to the inflammatory state and phenotype of the parent cell, including marked alterations in the AA pathway [101,102]. Importantly, EVs generated by cells within the cardiac microenvironment—including macrophages, mesenchymal stromal cells, and cardiomyoblasts—can carry diverse oxylipins together with functional enzymes required for their synthesis, suggesting that EVs may function as mobile sources of lipid mediators capable of modifying inflammatory signaling between cardiac cells [99]. Among these mediators, prostaglandins, leukotrienes, thromboxanes, and related AA metabolites have potent effects on vascular tone, platelet activation, inflammation, endothelial function, and thrombosis, processes that are central to atherosclerosis, myocardial ischemia, and other cardiovascular diseases [101]. Platelet-derived EVs are particularly relevant because activated platelets release EVs containing bioactive cargo and AA-derived mediators, providing a mechanism through which platelet activation can influence neighboring vascular and immune cells and amplify inflammatory and thrombotic signaling [101]. Consequently, changes in EV-associated AA and eicosanoid metabolism may provide an important link between cellular lipid metabolism and intercellular communication during cardiovascular disease, potentially contributing to endothelial dysfunction, vascular inflammation, atherogenesis, thrombosis, myocardial injury, and adverse cardiac remodeling [102,103].

## 3. EVs as Therapeutic Opportunities

The genesis and composition of EVs with native lipid bilayers, and thus high biocompatibility, makes them potentially attractive for drug delivery [14,15,16,17,18,19,29,34,37,104]. Particularly, the possibility to tailor the phospholipid/sphingolipid content and raft organization such that EVs are preferentially taken up by diseased cardiovascular tissues requires further studies [14,15,17,18,26,29,32,37]. There are efforts underway to generate hybrid vesicles of natural EVs and synthetic lipids, to allow controlled loading with drugs as well as targeting to specific tissues to treat myocardial infarction, heart failure, and vascular inflammation (Table 2) [14,15,17,18,19,29,34,37,105,106]. However, so far, the specific targeting of EVs has proven challenging due to missing biomarkers that are specific to the target organ. In preclinical models, EVs from stem cells improved cardiac function by reducing inflammation, enhancing angiogenesis, and limiting fibrosis and remodeling [15,42,107,108]. These beneficial effects are often attributed to miRNAs and proteins; however, lipid cargo likely modulates membrane fusion, receptor engagement, and signaling microdomains in recipient cardiovascular cells [14,15,17,18]. Specific studies on how the lipid composition of sEVS changes the function and targeting to tissues and cells are needed to clarify the question how lipids influence sEV behavior. Bioengineering EV membranes allows precise manipulation of their lipidomic array to control biological behavior and improve therapeutic delivery [29]. There are two general strategies currently explored to leverage sEVs as therapeutic tools: one is to create hybrid liposome-EV vesicles and the other is to decorate EVs with functional lipid moieties [29]. Surface modification using cardiac homing peptides conjugated to EV lipids extends sEV’s lifetime in myocardial tissue and improves functional outcomes after intravenous injection [109]. This modified-lipid approach is faster and more selective than traditional bio-combination methods, effectively changing surface epitope density regardless of the parent cell source [110]. Optimizing lipid composition or employing encapsulation strategies will be essential for increasing EV longevity and therapeutic efficacy in CVD patients [111]. Despite rapid progress in lipid identification and a beginning of mechanistic understanding, the clinical translation still faces major barriers, including the heterogeneous nature of EV subtypes and lack of biomarkers for EV classification, non-standardized isolation and quantification of EVs across studies, limited in vivo targeting specificity, partly due to the lack of specific EV type markers, and incomplete understanding of how specific lipid species influence safety, immunogenicity, and therapeutic efficacy [14,15,16,17,18,19,32,33,34,104]. Thus, large, well-controlled clinical studies integrating standardized EV lipidomics into CVD trials remain scarce.

**Table 2:** Overview of EV applications in CVD.

CVD Application	EV Source/Engineering	Key Lipid/Membrane Role	Citations
Myocardial Infarction	CVD Application	Lipid bilayer protects cargo; surface modification improves cardiac targeting	Chen 2024 [107] Li 2025 [112] Saludas 2021 [106]
Atherosclerosis	Myocardial Infarction	Lipid-raft-associated cargo transfer modulates smooth muscle cell phenotype	Ramasubramanian 2022 [113] Nguyen 2026 [105]
Ischemia/Reperfusion	Atherosclerosis	Membrane lipid optimization enhances stability and retention	Fu 2024 [111] Zhang 2022 [109]
Heart Failure	Ischemia/ Reperfusion	Lipid composition influences low immunogenicity and biocompatibility	Saludas 2021 [106] McDonald 2024 [108]

## 4. State of the Field and Future Directions

Much of the interest in EVs as intercellular communicators and therapeutic opportunities has largely been focused on protein and RNA composition [21,22,23,24,25]. These biases are likely due to workflows for the characterization of these molecules being more well established than those for lipids. However, dedicated efforts directed towards the improvement of lipidomic techniques have paved the way for more scientists to begin producing quality data. Despite these advancements, the resolution of lipid species still remains difficult, and lipids present an interesting problem in high-quality characterization. Beyond the lipid class, individual lipid species are extremely diverse based on the degree of saturation of their associated fatty acids. Identification of the precise location of double bonds is critical to understanding how a lipid impacts membrane dynamics, though mass spectrometry often does not provide clear double bond identifications. Integration of other methods, such as ion mobility and ozone-induced dissociation, can aid in distinguishing bond locations [114]. Additionally, major gaps in structural annotation and the limited availability of internal standards also make quantitative lipidomics challenging [115]. Many of these problems are being approached by the Lipidomics Standards Initiative, LIPID MAPS, and Metabolomics Standards Initiative to begin unifying lipidomic research, and researchers are encouraged to adhere to their suggestions to ensure data is accurate and reproducible [34,114,115].

Regarding contamination, EVs and lipoproteins share a large overlap in their physiochemical properties, which cannot be readily separated through common isolation methods such as ultracentrifugation and size-exclusion chromatography [116]. Inclusion of affinity filtration steps has been demonstrated to remove lipoproteins from EV samples without significant impacts to morphology or particle counts [117]. EVs isolated from the blood often carry a corona of common blood proteins, including lipoproteins, which can persist even with careful isolation techniques [116]. These coronae can have synergistic effects when associated with EVs. For instance, corona-intact EVs enhance angiogenesis in a manner that soluble factors or corona-depleted EVs alone cannot [118]. Based on this information, exactly what can be considered a contaminant versus what may be critical to a certain EV function requires more research.

Heterogeneity amongst EVs, even those from the same cell type and isolation event, provides an interesting challenge in the overall classification of their function. For instance, sEVs from cardiac progenitor cells show distinctions in size, cargo, and targeting ability [36]. Use of canonical EV markers typically associated with exosomes, such as tetraspanins CD9 and CD63, may initially seem appealing targets for immunoprecipitation to generate a homogeneous population for experimentation [116]. Experiments in HeLa cells indicate that both exosomes and ectosomes may be captured through such an approach [119]. Due to their differing routes of release and molecular content, the function of each sub-population is likely to vary, and co-precipitation would not allow the thorough interrogation of each individually. However, attempting to subdivide EVs based on their marker expression is still likely useful in determining functions of specific EVs sub-populations, and researchers should reference MISEV guidelines for how best to report which EVs are being investigated [116].

EVs are readily isolated from plasma, and thus their potential use as non-invasive diagnostic tools comes to mind. Several impediments for such use become apparent with further interrogation. The overall presence of non-blood cell-derived EVs within plasma is estimated to be extremely low, and isolation of tissue-specific EVs from such a complex sample remains difficult through affinity-based measures due to non-specific binding and isoform variation [34]. To begin understanding exactly which types of tissue-derived EVs enter circulation versus those that are taken up in a more paracrine manner will require careful experimentation. One possible route of investigation could involve the co-culture of cells from tissues commonly associated with one another, such as adipocytes and vascular endothelial cells, with specific tags to identify which cells release which EVs. A comparison of EVs isolated from each cell type in independent culture to those remaining in the media following co-culture could help discern which EVs are likely to enter the circulation. As organoid systems continue to develop, such an approach could offer even more insight into the complex world of EV signaling.

Lastly, the therapeutic value of EVs is heavily impacted by non-specific targeting. When administered intravenously, EVs have been shown to accumulate in major blood-filtering organs, such as the liver and spleen [120]. Cholesterol appears to be the major driver of liver targeting, which when removed from common liposome formulations, allows other organs to be targeted [121]. This is another area where more research into the specific targeting of EVs from diverse tissues is needed, as synthetic EVs could be produced to mirror natural EVs with high tissue tropism. Other routes of EV administration have been tested in animals, such as direct intramuscular injection into infarcted areas following myocardial infarction [122]. Such an approach is unlikely to directly transition into the treatment of human disease but indicates that localized administration of EVs has the potential to circumvent tropism issues common to systemic application.

## 5. Conclusion and Outstanding Questions

In conclusion, EV lipidomic analyses revealed that vesicles carry distinct, actively sorted lipid signatures that often mirror their cells of origin and modulate the metabolic and inflammatory state of their recipient cells. In CVD, multi-omics and functional data support EVs as promising tools for non-invasive diagnosis, risk stratification, and targeted therapy, though direct lipid-focused CVD studies are still limited. The role of lipids in CVD beyond cholesterol and TG is currently not well understood. This lack of knowledge extends to EV studies. Proteins and various RNA species are well-investigated components of EVs due to their known abilities to actively change the cell status and the easily available methodologies for their characterization and detection. Despite facing challenges in their characterization, careful consideration of all lipids is needed to advance the understanding of CVD and EV biology. While some lipids have been directly recognized as signaling molecules (for example, eicosanoids, S1P, and phosphatidylserine), lipids with a more structural nature, such as sphingomyelin and phosphatidylcholine, have not been sufficiently investigated for their contribution to CVD initiation and progression. Addressing the following outstanding questions in regard to EV lipid cargo and function will accelerate the field and potentially enable the use of EVs as therapeutic tools:

1. Do lipids contribute to specific targeting of EVs, and what is the mechanism?
2. How is the lipid composition of EVs regulated?
3. What are specific biomarkers for different EV subclasses?

Additionally, protein–lipid interactions are crucial to the normal function of cell membranes, and the evidence presented here suggests that changes in lipid composition of cardiovascular organs and EVs may disrupt normal protein function. While this review has exclusively focused on lipids, a holistic approach combining lipids, proteins, and genomic information may be critical to advancing the mechanistic understandings of general EV functionality and CVD progression. Progress toward clinical translation will depend on rigorous standardization of EV isolation and quantitative lipidomics, coupled with mechanistic work linking defined lipid species and membrane architectures to vascular and cardiac outcomes.

## Figures and Tables

**Table 1:** Summary of lipids present in EVs isolated from various sources with their proposed functions in cardiovascular disease.

Lipid Class	Notable Lipid Species	Functions and CVD Impact	Tissue Source of EV	Species and Models	Mass Spectrometry Method	Citations
Cholesterol	Cholesterol	• Lipid packing and acyl tail ordering • Immune modulation • Regulation of EV release	Mixed glial cells	Mouse	Non-specified MS methods	Chakraborty 2020 [40], Skotland 2020 [41], Abdullah 2021 [42]
Sphingomyelin	SM-16:0 SM-20:0 SM-22:0 SM-24:0 Sphingosine-1-phosphate	• Major membrane constituent • S1P disrupts calcium channel function in heart • Increased SM-16 correlated with all-cause mortality, longer chains inversely correlated	Plasma	Human	LC-MS/MS	Verderio 2018 [43], Pulli 2018 [44], Fretts 2024 [45]
Ceramide	Cer-16:0 Cer-24:0 Cer-24:1	• Critical for vascular relaxation • In atherosclerotic plaques • Excess Cer-16:0 induces apoptosis • Regulation of EV release, size, protein content	Adipocytes, Plasma, Oligo-dendrocytes	Mouse (3T3-L1, Oli-neu cells), Human-endothelial cells and patient sampling	UHPLC–ESI–Q-Exactive Plus MS/MS	Payet 2023 [35], Cantalupo 2020 [46], Meade 2023 [47], van de Wakker 2024 [36]
Glycosphingo-lipids	Gb3 GD1 GM1 HexCer (22:0, 24:0, 24:1) LacCer	• Variable membrane enrichment across cell types • Supports lipid raft and caveolae formation • Excess GSLs disrupt caveolar structure of endothelial cells	Adipocytes, ß-cells, Plasma	Human patient sampling, Mouse	Untargeted LC–MS/Q-TOF MS, targeted C–MS/MS with triple-quadrupole QTRAP MS	Schnaar 2022 [48], Stea 2025 [49], Mirza 2023 [50]
Phosphatidyl-serine	PS-40:6	• Cytosolic face of plasma membrane, externalized PS during apoptosis; supports tissue homeostasis • PS-enriched EVs promote clotting • Decreased expression in cardiomyocytes following MI, partially recovered by oral supplementation	Endothelial cells, Plasma, Epithelial cells	Human patient sampling, Mouse, Rat neonatal cardio-myocytes	shotgun tandem MS lipidomics	Shin 2020 [51], Kira 2023 [52], Das 2021 [53], Schumacher 2021 [54]
Phosphatidyl-choline	PC-(22:4/14:1), (14:0/22:6), (16:0/22.5), 42:10, 36:6, 40:9	• Membrane structure and shaping • Changes in PC species-level correlates with heart disease progression, particularly increased PC (22:4/14:1)	Plasma, Feces	Human, Mouse	LC–MS, automated ESI–tandem MS	McMahon 2015 [55], Rong 2023 [56], Timmerman 2022 [57], Kumar 2021 [37]

## Data Availability

No new data were created or analyzed in this study. Data sharing is not applicable to this article.

## References

[1] (2025). Global Burden of Cardiovascular Diseases and Risks 2023 Collaborators. Global, Regional, and National Burden of Cardiovascular Diseases and Risk Factors in 204 Countries and Territories, 1990–2023. J. Am. Coll. Cardiol..

[2] Choi R.H., Tatum S.M., Symons J.D., Summers S.A., Holland W.L. (2021). Ceramides and other sphingolipids as drivers of cardiovascular disease. Nat. Rev. Cardiol..

[3] Zhang H., Tan J., Suárez Y., Fernández-Hernando C. (2026). Emerging role of ceramides and other sphingolipids in atherosclerosis. Atherosclerosis.

[4] Yamaguchi A., Botta E., Holinstat M. (2022). Eicosanoids in inflammation in the blood and the vessel. Front. Pharmacol..

[5] Sherratt S.C., Mason R.P., Libby P., Bhatt D.L. (2024). “A Time to Tear Down and a Time to Mend”: The Role of Eicosanoids in Atherosclerosis. Arterioscler. Thromb. Vasc. Biol..

[6] Huang H., Ye G., Lai S.-Q., Zou H.-X., Yuan B., Wu Q.-C., Wan L., Wang Q., Zhou X.-L., Wang W.-J. (2021). Plasma Lipidomics Identifies Unique Lipid Signatures and Potential Biomarkers for Patients with Aortic Dissection. Front. Cardiovasc. Med..

[7] Jensen P.N., Fretts A.M., Hoofnagle A.N., McKnight B., Howard B.V., Umans J.G., Sitlani C.M., Siscovick D.S., King I.B., Sotoodehnia N. (2022). Circulating ceramides and sphingomyelins and the risk of incident cardiovascular disease among people with diabetes: The strong heart study. Cardiovasc. Diabetol..

[8] Sindhu S., Leung Y.H., Arefanian H., Madiraju S.R.M., Al-Mulla F., Ahmad R., Prentki M. (2021). Neutral sphingomyelinase-2 and cardiometabolic diseases. Obes. Rev..

[9] Morita S.-Y. (2024). Phospholipid biomarkers of coronary heart disease. J. Pharm. Health Care Sci..

[10] Rivas Serna I.M., Sitina M., Stokin G.B., Medina-Inojosa J.R., Lopez-Jimenez F., Gonzalez-Rivas J.P., Vinciguerra M. (2021). Lipidomic Profiling Identifies Signatures of Poor Cardiovascular Health. Metabolites.

[11] Calder P.C. (2020). Eicosanoids. Essays Biochem..

[12] Kolawole O.R., Kashfi K. (2025). Reprogramming Inflammation: Mechanisms and Therapeutic Targeting of Eicosanoids and Pro-Resolving Mediators. Eur. J. Pharmacol..

[13] Chistyakov D.V., Chistyakov V.V., Sergeeva M.G. (2025). Oxylipins in Atherosclerosis: Their Role in Inflammation, Diagnosis, and Therapeutic Perspectives. Int. J. Mol. Sci..

[14] Cappucci I.P., Tremoli E., Zavan B., Ferroni L. (2025). Circulating Extracellular Vesicles in Cardiovascular Disease. Int. J. Mol. Sci..

[15] Chen M., Wu Y., Chen C. (2025). Extracellular Vesicles as Emerging Regulators in Ischemic and Hypertrophic Cardiovascular Diseases: A Review of Pathogenesis and Therapeutics. Med. Sci. Monit..

[16] Davidson S.M., Boulanger C.M., Aikawa E., Badimon L., Barile L., Binder C.J., Brisson A., Buzas E., Emanueli C., Jansen F. (2023). Methods for the identification and characterization of extracellular vesicles in cardiovascular studies: From exosomes to microvesicles. Cardiovasc. Res..

[17] de Freitas R.C.C., Hirata R.D.C., Hirata M.H., Aikawa E. (2021). Circulating Extracellular Vesicles As Biomarkers and Drug Delivery Vehicles in Cardiovascular Diseases. Biomolecules.

[18] Han C., Yang J., Sun J., Qin G. (2022). Extracellular vesicles in cardiovascular disease: Biological functions and therapeutic implications. Pharmacol. Ther..

[19] Meng K., Meng F., Wu Y., Lin L. (2024). Multi-omics analysis identified extracellular vesicles as biomarkers for cardiovascular diseases. Talanta.

[20] Zhang Y., Lan M., Chen Y. (2024). Minimal Information for Studies of Extracellular Vesicles (MISEV): Ten-Year Evolution (2014–2023). Pharmaceutics.

[21] Hou J., Chen K.-X., He C., Li X.-X., Huang M., Jiang Y.-Z., Jiao Y.-R., Xiao Q.-N., He W.-Z., Liu L. (2024). Aged bone marrow macrophages drive systemic aging and age-related dysfunction via extracellular vesicle-mediated induction of paracrine senescence. Nat. Aging.

[22] Jeon O.H., Wilson D.R., Clement C.C., Rathod S., Cherry C., Powell B., Lee Z., Khalil A.M., Green J.J., Campisi J. (2019). Senescence cell-associated extracellular vesicles serve as osteoarthritis disease and therapeutic markers. JCI Insight.

[23] Kang M., Huang C.-C., Gajendrareddy P., Lu Y., Shirazi S., Ravindran S., Cooper L.F. (2022). Extracellular Vesicles from TNFα Preconditioned MSCs: Effects on Immunomodulation and Bone Regeneration. Front. Immunol..

[24] Senesi G., Guerricchio L., Ghelardoni M., Bertola N., Rebellato S., Grinovero N., Bartolucci M., Costa A., Raimondi A., Grange C. (2024). Extracellular vesicles from II trimester human amniotic fluid as paracrine conveyors counteracting oxidative stress. Redox Biol..

[25] Zhao H., Chen X., Hu G., Li C., Guo L., Zhang L., Sun F., Xia Y., Yan W., Cui Z. (2022). Small Extracellular Vesicles from Brown Adipose Tissue Mediate Exercise Cardioprotection. Circ. Res..

[26] Blandin A., Dugail I., Hilairet G., Ponnaiah M., Ghesquière V., Froger J., Ducheix S., Fizanne L., Boursier J., Cariou B. (2023). Lipidomic analysis of adipose-derived extracellular vesicles reveals specific EV lipid sorting informative of the obesity metabolic state. Cell Rep..

[27] Dorado E., Doria M.L., Nagelkerke A., McKenzie J.S., Maneta-Stavrakaki S., Whittaker T.E., Nicholson J.K., Coombes R.C., Stevens M.M., Takats Z. (2024). Extracellular vesicles as a promising source of lipid biomarkers for breast cancer detection in blood plasma. J. Extracell. Vesicles.

[28] Faria C.P., Ferreira B., Lourenço Á., Guerra I., Melo T., Domingues P., Domingues M.D.R.M., Cruz M.T., Sousa M.D.C. (2023). Lipidome of extracellular vesicles from *Giardia lamblia*. PLoS ONE.

[29] Ghadami S., Dellinger K. (2023). The lipid composition of extracellular vesicles: Applications in diagnostics and therapeutic delivery. Front. Mol. Biosci..

[30] Jiang Y., Zhu J., Liu J., Zhang H., Zhang P., Zhang L., Hou J., Tong P., Li Z., Zhao J. (2025). Deciphering the lipid profile: A quantitative lipidomic investigation into extracellular vesicles derived from human, ewe, and goat colostrum. J. Dairy Sci..

[31] Krokidis M.G., Pucha K.A., Mustapic M., Exarchos T.P., Vlamos P., Kapogiannis D. (2024). Lipidomic Analysis of Plasma Extracellular Vesicles Derived from Alzheimer’s Disease Patients. Cells.

[32] Lam S.M., Zhang C., Wang Z., Ni Z., Zhang S., Yang S., Huang X., Mo L., Li J., Lee B. (2021). A multi-omics investigation of the composition and function of extracellular vesicles along the temporal trajectory of COVID-19. Nat. Metab..

[33] Skotland T., Ekroos K., Llorente A., Sandvig K. (2025). Quantitative Lipid Analysis of Extracellular Vesicle Preparations: A Perspective. J. Extracell. Vesicles.

[34] Shami-Shah A., Travis B.G., Walt D.R. (2023). Advances in extracellular vesicle isolation methods: A path towards cell-type specific EV isolation. Extracell. Vesicles Circ. Nucleic Acids.

[35] Payet T., Valmori M., Astier J., Svilar L., Sicard F., Tardivel C., Ghossoub R., Martin J., Landrier J., Mounien L. (2023). Vitamin D Modulates Lipid Composition of Adipocyte-Derived Extracellular Vesicles Under Inflammatory Conditions. Mol. Nutr. Food Res..

[36] van de Wakker S.I., Bauzá-Martinez J., Arceo C.R., Manjikian H., Blok C.J.B.S., Roefs M.T., Willms E., Maas R.G.C., Pronker M.F., de Jong O.G. (2024). Size matters: Functional differences of small extracellular vesicle subpopulations in cardiac repair responses. J. Extracell. Vesicles.

[37] Kumar A., Sundaram K., Mu J., Dryden G.W., Sriwastva M.K., Lei C., Zhang L., Qiu X., Xu F., Yan J. (2021). High-fat diet-induced upregulation of exosomal phosphatidylcholine contributes to insulin resistance. Nat. Commun..

[38] Gupta P., Kadamberi I.P., Mittal S., Tsaih S., George J., Kumar S., Vijayan D.K., Geethadevi A., Parashar D., Topchyan P. (2022). Tumor Derived Extracellular Vesicles Drive T Cell Exhaustion in Tumor Microenvironment through Sphingosine Mediated Signaling and Impacting Immunotherapy Outcomes in Ovarian Cancer. Adv. Sci..

[39] Su H., Rustam Y.H., Masters C.L., Makalic E., McLean C.A., Hill A.F., Barnham K.J., Reid G.E., Vella L.J. (2021). Characterization of brain-derived extracellular vesicle lipids in Alzheimer’s disease. J. Extracell. Vesicles.

[40] Chakraborty S., Doktorova M., Molugu T.R., Heberle F.A., Scott H.L., Dzikovski B., Nagao M., Stingaciu L.-R., Standaert R.F., Barrera F.N. (2020). How cholesterol stiffens unsaturated lipid membranes. Proc. Natl. Acad. Sci. USA.

[41] Skotland T., Sagini K., Sandvig K., Llorente A. (2020). An emerging focus on lipids in extracellular vesicles. Adv. Drug Deliv. Rev..

[42] Abdullah M., Nakamura T., Ferdous T., Gao Y., Chen Y., Zou K., Michikawa M. (2021). Cholesterol Regulates Exosome Release in Cultured Astrocytes. Front. Immunol..

[43] Verderio C., Gabrielli M., Giussani P. (2018). Role of sphingolipids in the biogenesis and biological activity of extracellular vesicles. J. Lipid Res..

[44] Pulli I., Asghar M.Y., Kemppainen K., Törnquist K. (2018). Sphingolipid-mediated calcium signaling and its pathological effects. Biochim. Biophys. Acta Mol. Cell Res..

[45] Fretts A.M., Jensen P.N., Sitlani C.M., Hoofnagle A., Lidgard B., Umans J.G., Siscovick D.S., King I.B., Howard B.V., Cole S.A. (2024). Circulating Sphingolipids and All-Cause Mortality: The Strong Heart Family Study. J. Am. Heart Assoc..

[46] Cantalupo A., Sasset L., Gargiulo A., Rubinelli L., Del Gaudio I., Benvenuto D., Wadsack C., Jiang X.-C., Bucci M.R., Di Lorenzo A. (2020). Endothelial Sphingolipid De Novo Synthesis Controls Blood Pressure by Regulating Signal Transduction and NO via Ceramide. Hypertension.

[47] Meade R., Chao Y., Harroun N., Li C., Hafezi S., Hsu F.-F., Semenkovich C.F., Zayed M.A. (2023). Ceramides in peripheral arterial plaque lead to endothelial cell dysfunction. JVS Vasc. Sci..

[48] Schnaar R.L., Sandhoff R., Tiemeyer M., Kinoshita T., Varki A., Cummings R.D., Esko J.D., Stanley P., Hart G.W., Aebi M., Mohnen D., Kinoshita T., Packer N.H., Prestegard J.H. (2022). Essentials of Glycobiology.

[49] Stea D.M., D’Alessio A. (2025). Caveolae: Metabolic Platforms at the Crossroads of Health and Disease. Int. J. Mol. Sci..

[50] Mirza I., Haloul M., Hassan C., Masrur M., Mostafa A., Bianco F.M., Ali M.M., Minshall R.D., Mahmoud A.M. (2023). Adiposomes from Obese-Diabetic Individuals Promote Endothelial Dysfunction and Loss of Surface Caveolae. Cells.

[51] Shin H.-W., Takatsu H. (2020). Phosphatidylserine exposure in living cells. Crit. Rev. Biochem. Mol. Biol..

[52] Kira A., Tatsutomi I., Saito K., Murata M., Hattori I., Kajita H., Muraki N., Oda Y., Satoh S., Tsukamoto Y. (2023). Apoptotic extracellular vesicle formation via local phosphatidylserine exposure drives efficient cell extrusion. Dev. Cell.

[53] Das K., Keshava S., Ansari S.A., Kondreddy V., Esmon C.T., Griffin J.H., Pendurthi U.R., Rao L.V.M. (2021). Factor VIIa induces extracellular vesicles from the endothelium: A potential mechanism for its hemostatic effect. Blood.

[54] Schumacher D., Curaj A., Staudt M., Cordes F., Dumitraşcu A.R., Rolles B., Beckers C., Soppert J., Rusu M., Simsekyilmaz S. (2021). Phosphatidylserine Supplementation as a Novel Strategy for Reducing Myocardial Infarct Size and Preventing Adverse Left Ventricular Remodeling. Int. J. Mol. Sci..

[55] McMahon H.T., Boucrot E. (2015). Membrane curvature at a glance. J. Cell Sci..

[56] Rong J., He T., Zhang J., Bai Z., Shi B. (2023). Serum lipidomics reveals phosphatidylethanolamine and phosphatidylcholine disorders in patients with myocardial infarction and post-myocardial infarction-heart failure. Lipids Health Dis..

[57] Timmerman N., Waissi F., Dekker M., de Borst G.J., van Bennekom J., de Winter R.J., Hilvo M., Jylhä A., Pasterkamp G., de Kleijn D.P.V. (2022). Ceramides and phospholipids in plasma extracellular vesicles are associated with high risk of major cardiovascular events after carotid endarterectomy. Sci. Rep..

[58] Rai A., Huynh K., Cross J., Poh Q.H., Fang H., Claridge B., Duong T., Duarte C., Shaw J.E., Marwick T.H. (2025). Multi-omics identify hallmark protein and lipid features of small extracellular vesicles circulating in human plasma. Nat. Cell Biol..

[59] Graham T.R., Kozlov M.M. (2010). Interplay of proteins and lipids in generating membrane curvature. Curr. Opin. Cell Biol..

[60] Harayama T., Riezman H. (2018). Understanding the diversity of membrane lipid composition. Nat. Rev. Mol. Cell Biol..

[61] Burrello J., Biemmi V., Dei Cas M., Amongero M., Bolis S., Lazzarini E., Bollini S., Vassalli G., Paroni R., Barile L. (2020). Sphingolipid composition of circulating extracellular vesicles after myocardial ischemia. Sci. Rep..

[62] Qian X., Jia H., Wang J., He S., Yu M., Feng X., Gong Q., An Y., Wang X., Shi N. (2024). Circulating palmitoyl sphingomyelin levels predict the 10-year increased risk of cardiovascular disease death in Chinese adults: Findings from the Da Qing Diabetes Study. Cardiovasc. Diabetol..

[63] Keerthikumar S., Chisanga D., Ariyaratne D., Al Saffar H., Anand S., Zhao K., Samuel M., Pathan M., Jois M., Chilamkurti N. (2016). ExoCarta: A Web-Based Compendium of Exosomal Cargo. J. Mol. Biol..

[64] Bhat O.M., Mir R.A., Nehvi I.B., Wani N.A., Dar A.H., Zargar M.A. (2024). Emerging role of sphingolipids and extracellular vesicles in development and therapeutics of cardiovascular diseases. Int. J. Cardiol. Heart Vasc..

[65] Phan F., Bourron O., Foufelle F., Le Stunff H., Hajduch E. (2024). Sphingosine-1-phosphate signalling in the heart: Exploring emerging perspectives in cardiopathology. FEBS Lett..

[66] Wang N., Li J.-Y., Zeng B., Chen G.-L. (2023). Sphingosine-1-Phosphate Signaling in Cardiovascular Diseases. Biomolecules.

[67] Polzin A., Dannenberg L., Benkhoff M., Barcik M., Keul P., Ayhan A., Weske S., Ahlbrecht S., Trojovsky K., Helten C. (2023). Sphingosine-1-phosphate improves outcome of no-reflow acute myocardial infarction via sphingosine-1-phosphate receptor 1. ESC Heart Fail..

[68] Donoso-Quezada J., Ayala-Mar S., González-Valdez J. (2021). The role of lipids in exosome biology and intercellular communication: Function, analytics and applications. Traffic.

[69] Polzin A., Dannenberg L., Benkhoff M., Barcik M., Helten C., Mourikis P., Ahlbrecht S., Wildeis L., Ziese J., Zikeli D. (2023). Revealing concealed cardioprotection by platelet Mfsd2b-released S1P in human and murine myocardial infarction. Nat. Commun..

[70] Bernáth-Nagy D., Kalinyaprak M.S., Giannitsis E., Ábrahám P., Leuschner F., Frey N., Krohn J.B. (2024). Circulating extracellular vesicles as biomarkers in the diagnosis, prognosis and therapy of cardiovascular diseases. Front. Cardiovasc. Med..

[71] Skotland T., Llorente A., Sandvig K. (2023). Lipids in Extracellular Vesicles: What Can Be Learned about Membrane Structure and Function?. Cold Spring Harb. Perspect. Biol..

[72] Trajkovic K., Hsu C., Chiantia S., Rajendran L., Wenzel D., Wieland F., Schwille P., Brügger B., Simons M. (2008). Ceramide triggers budding of exosome vesicles into multivesicular endosomes. Science.

[73] Menck K., Sönmezer C., Worst T.S., Schulz M., Dihazi G.H., Streit F., Erdmann G., Kling S., Boutros M., Binder C. (2017). Neutral sphingomyelinases control extracellular vesicles budding from the plasma membrane. J. Extracell. Vesicles.

[74] Bockus L.B., Jensen P.N., Fretts A.M., Hoofnagle A.N., McKnight B., Sitlani C.M., Siscovick D.S., King I.B., Psaty B.M., Sotoodehnia N. (2023). Plasma Ceramides and Sphingomyelins and Sudden Cardiac Death in the Cardiovascular Health Study. JAMA Netw. Open.

[75] Zhang L., Zhang Y., Ding Y., Jin T., Song Y., Li L., Wang X., Zeng Y. (2025). Cer(d18:1/16:0) as a biomarkers for acute coronary syndrome in Chinese populations. Sci. Rep..

[76] Rubinelli L., Manzo O.L., Sungho J., Del Gaudio I., Bareja R., Marino A., Palikhe S., Di Mauro V., Bucci M., Falcone D.J. (2025). Suppression of endothelial ceramide de novo biosynthesis by Nogo-B contributes to cardiometabolic diseases. Nat. Commun..

[77] Peche V.S., Pietka T.A., Jacome-Sosa M., Samovski D., Palacios H., Chatterjee-Basu G., Dudley A.C., Beatty W., Meyer G.A., Goldberg I.J. (2023). Endothelial cell CD36 regulates membrane ceramide formation, exosome fatty acid transfer and circulating fatty acid levels. Nat. Commun..

[78] Huang S., Abutaleb K., Mishra S. (2024). Glycosphingolipids in Cardiovascular Disease: Insights from Molecular Mechanisms and Heart Failure Models. Biomolecules.

[79] Jiang C., Tan M., Lai L., Wang Y., Chen Z., Xie Q., Li Y. (2024). Inhibiting glycosphingolipids alleviates cardiac hypertrophy by reducing reactive oxygen species and restoring autophagic homeostasis. Front. Pharmacol..

[80] Skalska M.E., Durak-Kozica M., Stepien E.L. (2026). ToF-SIMS revealing sphingolipids composition in extracellular vesicles and paternal β-cells after persistent hyperglycemia. Talanta.

[81] Düsing P., Heinrich N.N., Al-Kassou B., Gutbrod K., Dörmann P., Nickenig G., Jansen F., Zietzer A. (2023). Analysis of circulating ceramides and hexosylceramides in patients with coronary artery disease and type II diabetes mellitus. BMC Cardiovasc. Disord..

[82] Kulkarni H., Meikle P.J., Mamtani M., Weir J.M., Barlow C.K., Jowett J.B., Bellis C., Dyer T.D., Johnson M.P., Rainwater D.L. (2013). Plasma lipidomic profile signature of hypertension in Mexican American families: Specific role of diacylglycerols. Hypertension.

[83] de Oliveira Campos J., Pereira J.G., Simões-Alves A.C., Vitalis O., Vega N., Chikh K., Leandro C.G., Pirola L., Costa-Silva J.H. (2026). Inverse association between circulating hexosylceramides and cardiometabolic risk factors in adult women with overweight-obesity. Nutr. Res..

[84] Groß R., Reßin H., von Maltitz P., Albers D., Schneider L., Bley H., Hoffmann M., Cortese M., Gupta D., Deniz M. (2024). Phosphatidylserine-exposing extracellular vesicles in body fluids are an innate defence against apoptotic mimicry viral pathogens. Nat. Microbiol..

[85] Das K., Keshava S., Mukherjee T., Wang J., Magisetty J., Kolesnick R., Pendurthi U.R., Rao L.V.M. (2023). Factor VIIa releases phosphatidylserine-enriched extracellular vesicles from endothelial cells by activating acid sphingomyelinase. J. Thromb. Haemost..

[86] Wang J., Yu X., Wang T., Cai W., Hua T., Duan J., Zhang X., Zhu Y., Yao L. (2023). Metabolic changes of glycerophospholipids during the reparative phase after myocardial infarction injury. Front. Cardiovasc. Med..

[87] Jafarzadeh A., Poorgholami M., Nemati M., Rezayati M.-T. (2011). High serum levels of rheumatoid factor and anti-phosphatidylserine antibody in patients with ischemic heart disease. Iran. J. Immunol..

[88] Vianello E., Ambrogi F., Kalousová M., Badalyan J., Dozio E., Tacchini L., Schmitz G., Zima T., Tsongalis G.J., Corsi-Romanelli M.M. (2024). Circulating perturbation of phosphatidylcholine (PC) and phosphatidylethanolamine (PE) is associated to cardiac remodeling and NLRP3 inflammasome in cardiovascular patients with insulin resistance risk. Exp. Mol. Pathol..

[89] Kvasnička A., Kotaška K., Friedecký D., Ježdíková K., Brumarová R., Hnát T., Kala P. (2024). Long-chain polyunsaturated fatty acid-containing phosphatidylcholines predict survival rate in patients after heart failure. Heliyon.

[90] Shoghli M., Sinisalo J., Lokki A.I., Lääperi M., Lokki M.-L., Hilvo M., Jylhä A., Tuomilehto J., Laaksonen R. (2025). Exploring the association between ceramide, phosphatidylcholine, and COPD prevalence and incidence: A FINRISK population-based cohort study. BMC Pulm. Med..

[91] Rapushi E., Aryal A., Yang T., Li Z., Wang X., Fan G.-C. (2026). Mitochondria-Derived Vesicles and Mitochondrial Extracellular Vesicles in Health and Cardiovascular Disease. Circ. Res..

[92] Dong Z., Liu S., Liu X., He D., Chu Q., Zhong L., Yu X., Fu X. (2025). Therapeutic mitochondrial transfer via mesenchymal stem cell-derived microvesicles for macrophage modulation in myocardial infarction complicated by diabetes. Biomaterials.

[93] Heyn J., Heuschkel M.A., Goettsch C. (2023). Mitochondrial-Derived Vesicles—Link to Extracellular Vesicles and Implications in Cardiovascular Disease. Int. J. Mol. Sci..

[94] Peng T., Xie Y., Sheng H., Wang C., Lian Y., Xie N. (2022). Mitochondrial-derived vesicles: Gatekeepers of mitochondrial response to oxidative stress. Free Radic. Biol. Med..

[95] Picca A., Guerra F., Calvani R., Coelho-Junior H.J., Bossola M., Landi F., Bernabei R., Bucci C., Marzetti E. (2020). Generation and Release of Mitochondrial-Derived Vesicles in Health, Aging and Disease. J. Clin. Med..

[96] Ali M.A., Gioscia-Ryan R., Yang D., Sutton N.R., Tyrrell D.J. (2023). Cardiovascular Aging: Spotlight on Mitochondria. Am. J. Physiol. Heart Circ. Physiol..

[97] Wei J., Zhang M., Wang X., Yang K., Xiao Q., Zhu X., Pan X. (2024). Role of Cardiolipin in regulating and treating atherosclerotic cardiovascular diseases. Eur. J. Pharmacol..

[98] Swain L., Bhave S., Qiao X., Reyelt L., Everett K.D., Awata J., Raghav R., Powers S.N., Sunagawa G., Natov P.S. (2024). Novel Role for Cardiolipin as a Target of Therapy to Mitigate Myocardial Injury Caused by Venoarterial Extracorporeal Membrane Oxygenation. Circulation.

[99] Ong-Meang V., Blanzat M., Savchenko L., Perquis L., Guardia M., Pizzinat N., Poinsot V. (2023). Extracellular Vesicles Produced by the Cardiac Microenvironment Carry Functional Enzymes to Produce Lipid Mediators In Situ. Int. J. Mol. Sci..

[100] Reinicke M., Shamkeeva S., Hell M., Isermann B., Ceglarek U., Heinemann M.L. (2022). Targeted Lipidomics for Characterization of PUFAs and Eicosanoids in Extracellular Vesicles. Nutrients.

[101] Contursi A., Tacconelli S., Di Berardino S., De Michele A., Patrignani P. (2024). Platelets and extracellular vesicles in disease promotion via cellular cross-talk and eicosanoid biosynthesis. Prostaglandins Other Lipid Mediat..

[102] Zisser L., Binder C.J. (2024). Extracellular Vesicles as Mediators in Atherosclerotic Cardiovascular Disease. J. Lipid Atheroscler..

[103] Badimon L., Padro T., Arderiu G., Vilahur G., Borrell-Pages M., Suades R. (2022). Extracellular vesicles in atherothrombosis: From biomarkers and precision medicine to therapeutic targets. Immunol. Rev..

[104] Singh M., Tiwari P.K., Kashyap V., Kumar S. (2025). Proteomics of Extracellular Vesicles: Recent Updates, Challenges and Limitations. Proteomes.

[105] Nguyen T.H., Huynh N.T., Niego B., Hagemeyer C.E., Xu R. (2026). Advances in Engineered Extracellular Vesicles for the Treatment of Atherosclerosis and Atherothrombosis—Next-Generation Cardiovascular Nanomedicine. Small Struct..

[106] Saludas L., Oliveira C.C., Roncal C., Ruiz-Villalba A., Prósper F., Garbayo E., Blanco-Prieto M.J. (2021). Extracellular Vesicle-Based Therapeutics for Heart Repair. Nanomaterials.

[107] Chen D.-X., Lu C.-H., Na N., Yin R.-X., Huang F. (2024). Endothelial progenitor cell-derived extracellular vesicles: The world of potential prospects for the treatment of cardiovascular diseases. Cell Biosci..

[108] McDonald J., Mohak S., Fabian Z. (2024). Stem Cell-Derived Extracellular Vesicles in the Treatment of Cardiovascular Diseases. Pharmaceutics.

[109] Zhang X., Wu Y., Cheng Q., Bai L., Huang S., Gao J. (2022). Extracellular Vesicles in Cardiovascular Diseases: Diagnosis and Therapy. Front. Cell Dev. Biol..

[110] Cui Z., Zhang L., Hu G., Zhang F. (2024). Extracellular Vesicles in Cardiovascular Pathophysiology: Communications, Biomarkers, and Therapeutic Potential. Cardiovasc. Toxicol..

[111] Fu E., Pan K., Li Z. (2024). Engineering extracellular vesicles for targeted therapeutics in cardiovascular disease. Front. Cardiovasc. Med..

[112] Li H., Wang L., Cheng H., Zhang Q., Wang S., Zhong W., He C., Wei Q. (2025). Unlocking the Potential of Extracellular Vesicles in Cardiovascular Disease. J. Cell. Mol. Med..

[113] Ramasubramanian L., Du S., Gidda S., Bahatyrevich N., Hao D., Kumar P., Wang A. (2022). Bioengineering Extracellular Vesicles for the Treatment of Cardiovascular Diseases. Adv. Biol..

[114] Kim K.-H., Yoo B.C. (2026). Decoding Membrane Lipids: Analytical Barriers and Technological Advances in Modern Lipidomics. Int. J. Mol. Sci..

[115] Fedorova M., Bendt A.K., Bertrand-Michel J., Fiehn O., Godzien J., Goracci L., Gruber F., Guan X.L., Han X., Holčapek M. (2026). A lipidomics roadmap: From basic research to societal challenges. Nat. Commun..

[116] Welsh J.A., Goberdhan D.C.I., O’DRiscoll L., Buzas E.I., Blenkiron C., Bussolati B., Cai H., Di Vizio D., Driedonks T.A.P., Erdbrügger U. (2024). Minimal information for studies of extracellular vesicles (MISEV2023): From basic to advanced approaches. J. Extracell. Vesicles.

[117] Jeon S., Kim Y., Shin S. (2026). Selective Lipoprotein Removal Enables High-Purity EV Isolation from Plasma via Aptamer-Based Mesh Filtration. Small.

[118] Wolf M., Poupardin R.W., Ebner-Peking P., Andrade A.C., Blöchl C., Obermayer A., Gomes F.G., Vari B., Maeding N., Eminger E. (2022). A functional corona around extracellular vesicles enhances angiogenesis, skin regeneration and immunomodulation. J. Extracell. Vesicles.

[119] Mathieu M., Névo N., Jouve M., Valenzuela J.I., Maurin M., Verweij F.J., Palmulli R., Lankar D., Dingli F., Loew D. (2021). Specificities of exosome versus small ectosome secretion revealed by live intracellular tracking of CD63 and CD9. Nat. Commun..

[120] Frolova L., Li I.T.S. (2022). Targeting Capabilities of Native and Bioengineered Extracellular Vesicles for Drug Delivery. Bioengineering.

[121] Su K., Shi L., Sheng T., Yan X., Lin L., Meng C., Wu S., Chen Y., Zhang Y., Wang C. (2024). Reformulating lipid nanoparticles for organ-targeted mRNA accumulation and translation. Nat. Commun..

[122] Qin D., Wang X., Pu J., Hu H. (2024). Cardiac cells and mesenchymal stem cells derived extracellular vesicles: A potential therapeutic strategy for myocardial infarction. Front. Cardiovasc. Med..

